# RNAi Identified the Potential Functions of Actin-like Protein in the Growth Performance of *Macrobrachium nipponense*

**DOI:** 10.3390/ijms27020893

**Published:** 2026-01-15

**Authors:** Shubo Jin, Jinyu Lin, Hongtuo Fu, Yiwei Xiong, Hui Qiao, Wenyi Zhang, Sufei Jiang

**Affiliations:** 1 Wuxi Fisheries College, Nanjing Agricultural University, Wuxi 214081, China; jinsb@ffrc.cn (S.J.); 13129105669@163.com (J.L.); fuht@ffrc.cn (H.F.); qiaoh@ffrc.cn (H.Q.); 2 Key Laboratory of Freshwater Fisheries and Germplasm Resources Utilization, Ministry of Agriculture and Rural Affairs, Freshwater Fisheries Research Center, Chinese Academy of Fishery Sciences, Wuxi 214081, China; xiongyw@ffrc.cn

**Keywords:** oriental river prawn, muscle histology, genetic improvement, marker-assisted selection, SNPs

## Abstract

*Macrobrachium nipponense* is an important commercial freshwater prawn species in China. Since larger individuals command higher market value, there is a pressing need to identify growth-related genes and single-nucleotide polymorphisms (SNPs) to facilitate genetic improvement in this species. Previous studies have suggested a potentially regulatory role of an actin-like protein (*ACTL*) in the growth of *M. nipponense*. Therefore, the present study aimed to functionally characterize the role of *ACTL* in growth and identify growth-associated SNPs within this gene. The open reading frame of *Mn-ACTL* is 1131 bp, encoding a protein with 377 amino acids. Blastx and phylogenetic analyses indicated that *Mn-ACTL* shares a close evolutionary relationship with orthologs from *Macrobrachium rosenbergii* and *Palaemon carinicauda*. The highest expression level of *Mn-ACTL* in muscle tissue detected by qPCR suggested its potential involvement in growth regulation. RNA interference experiments showed that prawns injected with *dsGFP* exhibited larger body sizes than those injected with *dsACTL*, indicating that knockdown of *Mn-ACTL* expression inhibits growth performance in *M. nipponense*. Furthermore, muscle tissue from the *dsACTL*-injected group displayed looser myofibril packing, visibly eroded areas, and increased sarcomere spacing. Collectively, these results demonstrated that *ACTL* positively regulates growth in *M. nipponense*. Additionally, the T allele at locus S28_17149891 and the G allele at locus S28_17145758 were significantly associated with growth traits (*p* < 0.05). In conclusion, this study confirmed the positive regulatory role of *ACTL* in growth and identified growth-associated SNPs in *M. nipponense*, providing valuable insights for breeding new varieties with enhanced growth performance in this species.

## 1. Introduction

Growth performance is a key objective in the genetic improvement of aquatic animals. Larger individuals offer greater economic benefits compared to smaller ones. Actin plays a regulatory role in the growth and development of various human cancers, including gastric [1], breast [2], and pancreatic cancers [3]. Beyond oncogenesis, actin is considered a candidate gene associated with individual growth performance in crustaceans [4,5]. Its functions have been well-characterized in the giant freshwater prawn, *Macrobrachium rosenbergii* [6]. Subsequently, growth-related single-nucleotide polymorphisms (SNPs) have been identified within this gene, facilitating genetic improvement through marker-assisted selection in this species [7].

Transcriptome profiling revealed that actin-like (*ACTL*) was significantly upregulated in the muscle of fast-growing prawns compared to slow-growing individuals, suggesting its critical role in the regulation of growth performance in *M. nipponense* [8]. Actin is a ubiquitous eukaryotic protein and a major constituent of the cytoskeleton in all cell types with several paralogs [9,10]. They play critical roles in diverse cellular processes, including muscle contraction, cell shape maintenance, adhesion, motility, intracellular trafficking, and cell division, which are essential for overall body growth [11,12,13,14].

The oriental river prawn, *Macrobrachium nipponense*, is an important commercial freshwater species widely distributed in China and other Asian countries [15,16,17,18,19]. In China, the annual production of this species is approximately 230,000 metric tons, accounting for 5.72% of the total freshwater prawn production and generating substantial economic benefits [20]. There is an urgent need to identify growth-related genes and single-nucleotide polymorphisms (SNPs) to facilitate genetic enhancement of growth performance in *M. nipponense*. In our previous study, a genome-wide association study was conducted to screen for growth-related genes and SNPs in *M. nipponense* [8]. However, to the best of our knowledge, no studies have yet reported functional analyses of growth-related genes in this species.

In the present study, we aimed to investigate the potential roles of *ACTL* in regulating growth performance in *M. nipponense* using quantitative PCR (qPCR) and RNA interference (RNAi). Additionally, growth-related SNPs were identified in this gene through PCR amplification. This study enhances the understanding of the molecular mechanisms underlying growth and contributes to the development of new varieties with improved growth traits via marker-assisted selection.

## 2. Results

### 2.1. Sequence Analysis

**Figure 1:**
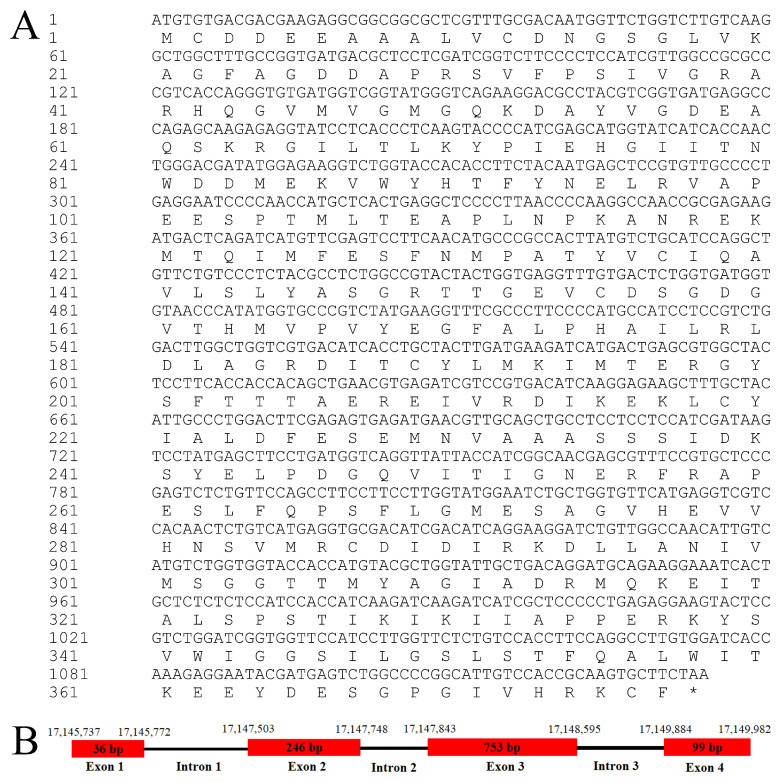
(**A**) The open reading frame sequence of the *ACTL* gene. Both nucleotide and deduced amino acid sequences are presented in the 5′ to 3′ direction. A single uppercase letter represents the amino acid code in the deduced amino acid sequence. The methionine initiation codon (ATG) and termination codon (TAA, indicated by an asterisk) are explicitly annotated. (**B**) The genome structure of the *ATCL* gene. The number indicates the start and end positions of each exon.

**Figure 2:**
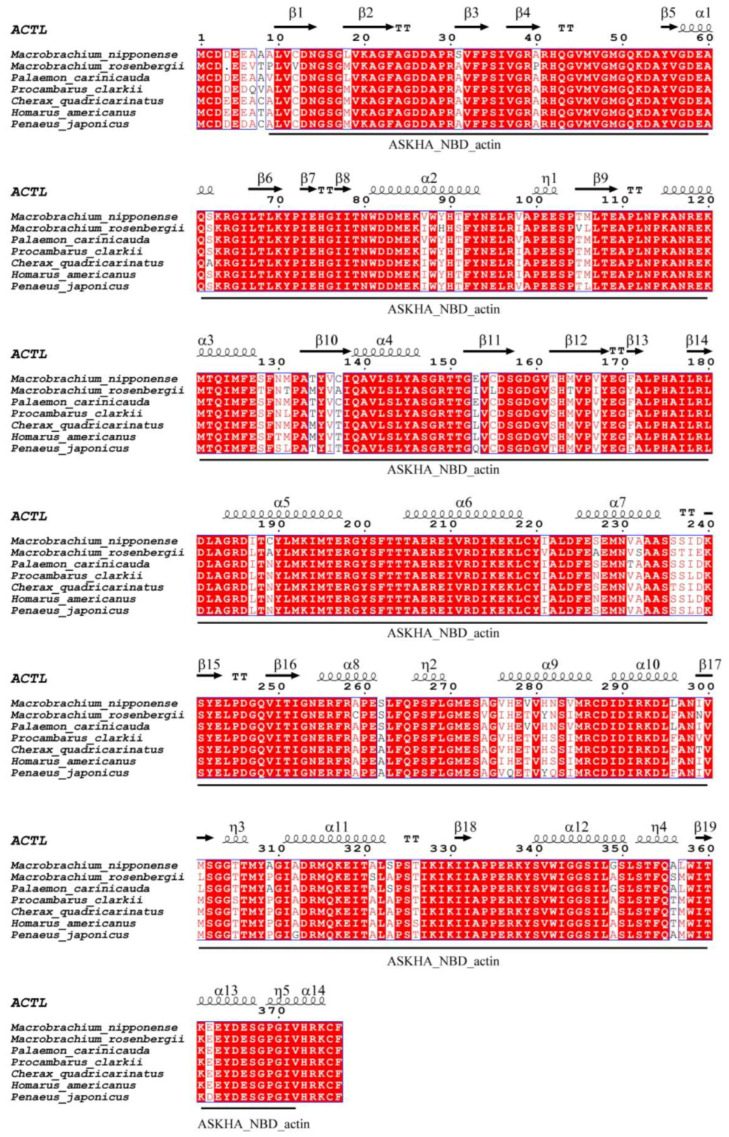
Sequence alignment and structural information of the *ACTL* protein from *M. nipponense*. α: α-helix, β: β-sheet, TT: β-bend, η: 310-helix. Red shades indicated that the amino acid sequences were identical across different species, while white shades indicates that the amino acid sequences varied among species. The black font color indicated differences in amino acids among the different species.

**Figure 3:**
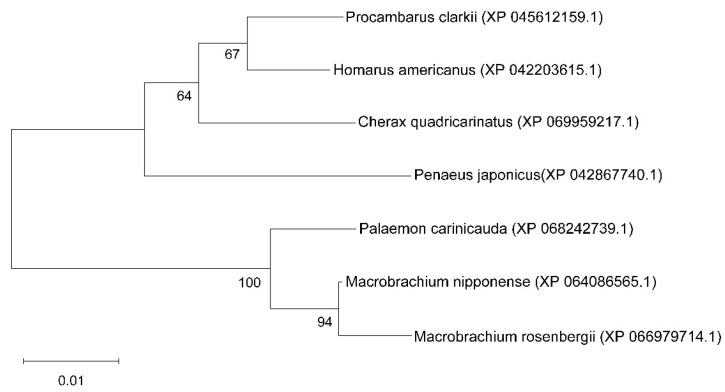
Phylogenetic tree analysis of *ACTL* protein in crustaceans.

The open reading frame (ORF) of *Mn-ACTL* annotated from the *M. nipponense* reference genome [21] was 1134 bp, encoding 377 amino acids from nucleotide position 17145737 to 17149982 of chromosome 28 (NCBI accession: CP062029) (Figure 1A). The genomic sequence of *Mn-ACTL* was 4246 bp, comprising four exons and three introns (Figure 1B). The theoretical *pI* and the protein molecular weight were 5.11 and 41,623 Da, respectively. The secondary structure of this protein comprises 14 α-helices, 19 β-sheets, 7 β-turns, and 5 310-helices. A conserved functional domain, ASKHA_NBD_actin, was predicted within the protein, spanning amino acid residues 8 to 372 (Figure 2). Additionally, the gene was found to be highly conserved among crustaceans. In a multiple sequence alignment involving seven crustacean species, 317 sites exhibited 100% sequence identity, accounting for approximately 84% of all amino acid residues (Figure 2). Blastx analysis against the NCBI database revealed that the *Mn-ACTL* amino acid sequence exhibited high identity (>92%) with its orthologs from other shrimp species. It shared the highest identity with *M. rosenbergii* (99.20%), followed by *Palaemon carinicauda* (98.41%) and *Cherax quadricarinatus* (93.37%). The maximum likelihood phylogenetic analysis revealed that the *Mn-ACTL* amino acid sequence is most closely related to that of *M. rosenbergii*, forming a clade with *P. carinicauda*. This observed phylogenetic relationship is consistent with the results from the sequence similarity alignment (Figure 3).

### 2.2. qPCR Analysis in Different Mature Tissues

**Figure 4:**
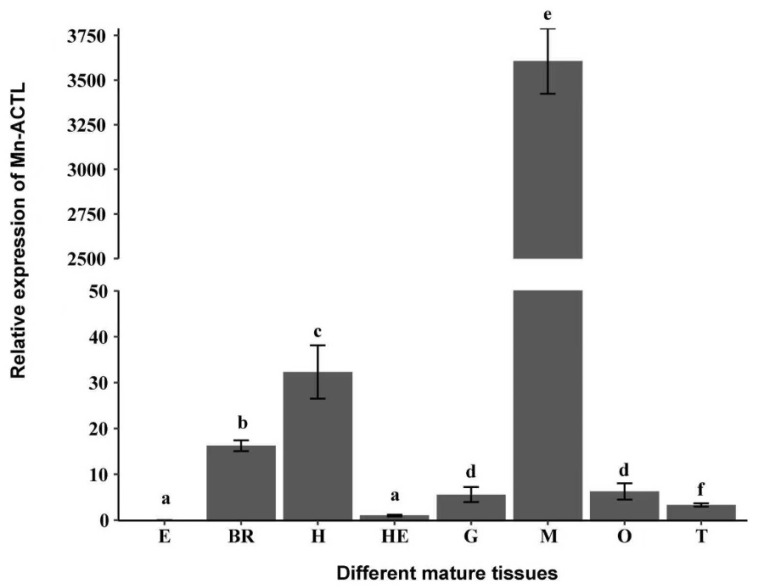
The relative expression levels of the *ACTL* gene in various mature tissues of *M. nipponense* were determined by qPCR. The *EIF* gene was used as an internal reference for normalization. Data were presented as the mean ± standard deviation (SD; n = 6). Significant differences in *ACTL* expression among different tissues were indicated by lowercase letters (*p* < 0.05). E, eyestalk; BR, brain; H, heart; HE, hepatopancreas; G, gill; M, muscle; O, ovary; T, testis.

The highest transcript level of *Mn-ACTL* was detected in muscle, which was significantly greater than in all other examined tissues (*p* < 0.05). Specifically, the expression in muscle was 3612.37-fold higher than that in the eyestalk. Moderately high expression was observed in the heart and brain, respectively, whereas transcript levels in the remaining tissues were negligible (Figure 4).

### 2.3. RNAi Analysis

**Figure 5:**
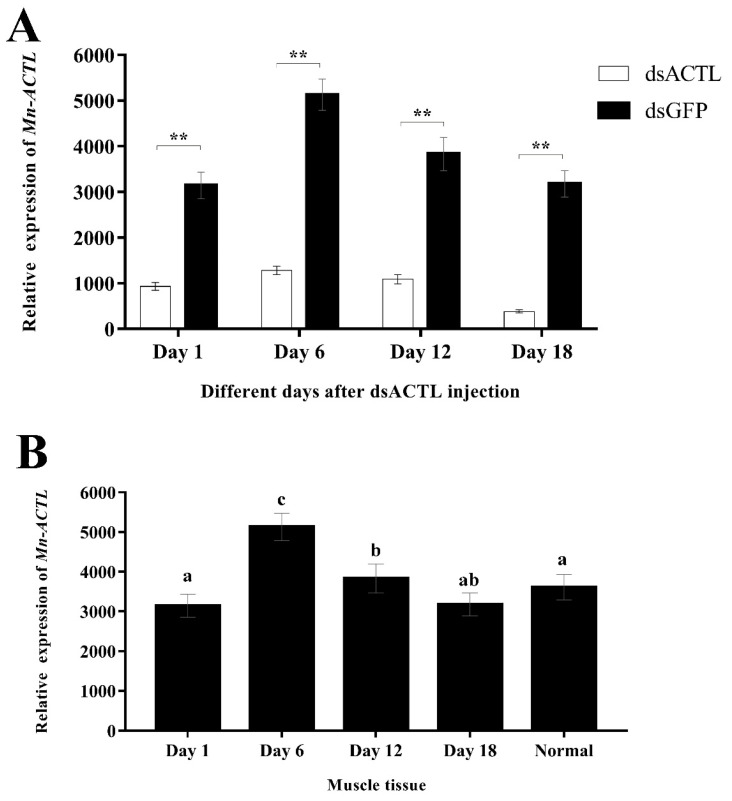
(**A**) The interference efficiency of *dsACTL* was monitored over a time course by qPCR. Gene expression data were normalized to the *EIF* reference gene and were expressed as the mean ± SD (n = 6). Asterisks (**) denote a highly significant difference (*p* < 0.01) between the *dsACTL* and *dsGFP* control groups at identical time points. (**B**) Comparison of Mn-ACTL expression in muscle tissue from *dsGFP*-injected and normal control groups. The “Normal” group represents untreated control groups. Lowercase letters indicated significant differences (*p* < 0.05).

Compared to the *dsGFP*-injected controls, the *dsACTL*-injected group showed a successful knockdown, with *Mn-ACTL* expression suppressed by 70.28% to 90.84% across the sampling days (Figure 5A). Additionally, *Mn-ACTL* expression was compared between muscle tissue from the *dsGFP*-injected group and normal (untreated) muscle (Figure 5B). Relative to the normal muscle control, expression levels in tissues sampled on days 1, 6, 12, and 18 were 0.87-, 1.42-, 1.06-, and 0.88-fold, respectively.

**Figure 6:**
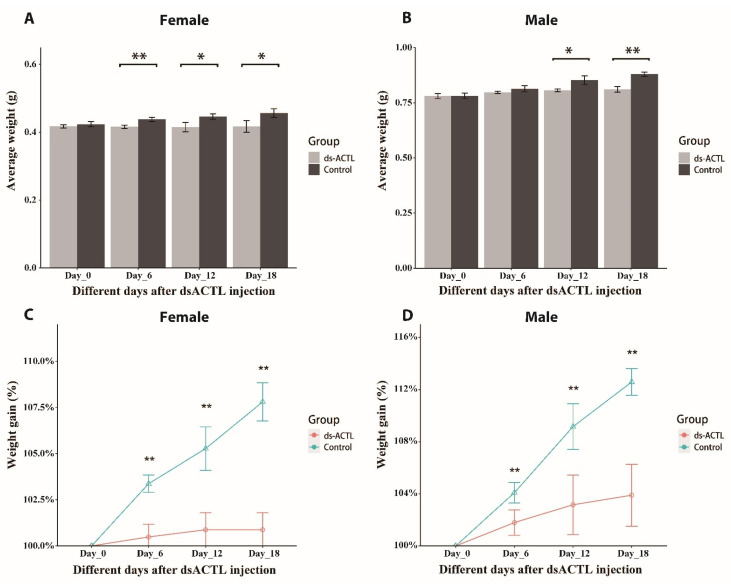
Effect of *dsACTL* injection on weight gain in *M. nipponense*. Body weight changes were monitored over time following injection with *dsACTL* or *dsGFP* (control). Significant differences between the *dsACTL* and *dsGFP* groups at the same time point are indicated by * (*p* < 0.05) and ** (*p* < 0.01). (**A**) Weight gain in females. (**B**) Weight gain in males. (**C**) Percentage increase in body mass for females. (**D**) Percentage increase in body mass for males.

The initial mean body weights of female prawns at day 0 were comparable between the *dsGFP*-injected control (0.424 ± 0.05 g) and the *dsACTL*-injected group (0.417 ± 0.05). By days 6, 12, and 18, the weights in the *dsGFP*-injected control reached 0.438 ± 0.06 g, 0.446 ± 0.06 g, and 0.457 ± 0.06 g, respectively. In contrast, the *dsACTL*-injected group exhibited lower weights of 0.419 ± 0.04 g, 0.421 ± 0.05 g, and 0.422 ± 0.05 g at the same time points (Figure 6A). A similar pattern was observed in males. The initial weights were 0.781 ± 0.11 g for the *dsGFP*-injected control and 0.781 ± 0.10 g for the *dsACTL*-injected group. Over time, the *dsGFP*-injected control attained weights of 0.814 ± 0.12 g, 0.855 ± 0.11 g, and 0.881 ± 0.13 g on days 6, 12, and 18, respectively. In contrast, the *dsACTL*-injected group exhibited markedly lower weights of 0.794 ± 0.11 g, 0.805 ± 0.11 g, and 0.811 ± 0.12 g at the same intervals (Figure 6B).

When expressed as percentage mass increase relative to day 0, female prawns in the *dsGFP*-injected control showed gains of 1.79%, 3.16%, and 3.89% on days 6, 12, and 18, respectively, which were significantly higher than the 0.48%, 0.88%, and 0.88% increases observed in the *dsACTL*-injected group (*p* < 0.01) (Figure 6C). Similarly, male prawns in the *dsGFP*-injected control displayed mass increases of 4.08%, 9.16%, and 12.59%, significantly greater than the 3.37%, 5.28%, and 7.81% increases in the *dsACTL*-injected group (*p* < 0.01) (Figure 6D).

On day 18 of the experiment, the daily weight gain rate (DWGR) and daily length gain rate (DLGR) in the *dsACTL*-injected group were significantly lower than those in the *dsGFP*-injected control for both female and male prawns (*p* < 0.05). Specifically, the female prawns in the *dsACTL*-injected group showed an 80.40% reduction in DWGR and a 27.50% reduction in DLGR compared to the controls. Similarly, the male prawns exhibited decreases of 57.91% in DWGR and 67.47% in DLGR.

### 2.4. Histological Observation

**Figure 7:**
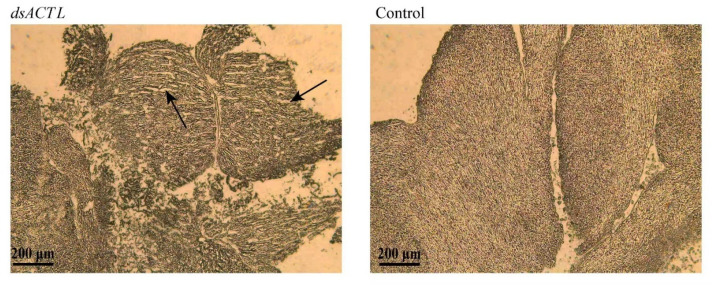
Histological changes of abdominal muscle tissue after 18 days of *dsACTL* and *dsGFP* injection. The area indicated by the arrow was the scattered erosion region in myofibrils.

Histological analysis of abdominal muscles on day 18 revealed distinct morphological differences between the groups (Figure 7). The prawns in *dsGFP*-injected control exhibited tightly arranged myofibrils and continuous sarcomeres, whereas the prawns in *dsACTL*-injected group displayed looser myofibril packing, evident eroded areas, and consequently, increased sarcomere spacing.

### 2.5. Identification of SNPs

A total of 18 SNPs were identified within the *Mn-ACTL* coding region. These loci exhibited observed heterozygosity (*Ho*) ranging from 0.074 to 0.418, expected heterozygosity (*He*) from 0.127 to 0.385, and polymorphism information content (*PIC*) from 0.097 to 0.989. All 18 sites were synonymous mutations (Table 1).

Among these 18 SNPs, two SNPs were identified to be significantly associated with growth traits of *M. nipponense* (false discovery ratio < 0.05). The locus S28_17145758 was pointed at the 8th amino acid of the gene, whereas the other locus S28_17149891 was localized at the 348th amino acid (Table 1).

A total of 91 individuals (45 females and 46 males) were successfully genotyped at the S28_17145758 locus (Table 2). Among the 91 genotyped individuals, those with the GG genotype exhibited significantly greater body weight and length than those with either the CC or CG genotype (*p* < 0.05), though GG female was absent among genotyped individuals. The CG genotype (n = 11 and 13, respectively) showed better growth than the CC genotype (*p* < 0.05), but inferior to the GG among males (*p* < 0.05).

A total of 86 individuals (44 females and 42 males) were successfully genotyped at the S28_17149891 locus (Table 2). The TT genotype was identified in 5 individuals, who showed the highest averages in both body weight and full length (*p* < 0.05). Better growth of the TT genotype was also confirmed when separately examined by sex (*p* < 0.05).

## 3. Discussion

Growth performance is a primary target for the genetic improvement of *M. nipponense*. Marker-assisted selection is a modern breeding technique that enhances selection accuracy and shortens the breeding cycle [22,23,24,25]. Consequently, there is a pressing need to identify growth-related genes and SNPs in *M. nipponense* to facilitate genetic improvement for superior growth. As a candidate gene, *ACTL* has been implicated in the regulation of growth performance in this species [8]. To this end, this study aimed to validate the regulatory role of *ACTL* on growth and to identify growth-associated SNPs within this gene, thereby contributing to the genetic improvement of *M. nipponense*.

To the best of our knowledge, the expression of actin genes has rarely been analyzed in aquatic animals. Northern blot analysis revealed that the actin gene was predominantly expressed in the muscle tissues of *M. rosenbergii*, while transcripts in the hepatopancreas were barely detectable. The expression of the actin gene varied during embryonic development, peaking at the zoea stage [6]. In the present study, the highest expression level of *Mn-ACTL* was observed in muscle tissue, consistent with the pattern observed in *M. rosenbergii*, suggesting that *Mn-ACTL* may be involved in regulating growth performance in *M. nipponense*.

RNAi has been widely employed as an established approach for the functional characterization of genes related to reproduction [26,27,28], growth [29] and immune defense [30,31,32] in *M. nipponense*. Here, we report for the first time the application of RNAi to investigate the role of the actin gene in regulating growth performance in *M. nipponense*. In the present study, injection of *dsACTL* significantly suppressed *Mn-ACTL* expression, demonstrating that the synthesized *dsACTL* effectively knocks down *Mn-ACTL* expression. The injection of *dsACTL* impaired growth performance in both sexes, as evidenced by reduced mass gain and disrupted sarcomere spacing. Collectively, these results indicated that *Mn-ACTL* plays a positive role in regulating growth performance in *M. nipponense*. The variation in *Mn-ACTL* expression within the *dsGFP* group across sampling days could reflect the influence of both endogenous biological rhythms and exogenous environmental cues.

Associations between SNPs and key traits have been documented in *M. nipponense*, regarding hypoxia resistance [33] and sexual maturation [34]. In the present study, a total of 18 SNP loci were identified within the coding region of the *ACTL* gene in *M. nipponense*. Two of these loci, S28_17145758 and S28_17149891, were found to be associated with growth performance. The identified potential molecular markers could facilitate the genetic improvement of growth traits through marker-assisted selection in this species.

## 4. Materials and Methods

### 4.1. Tissue Collection

A total of 268 healthy individuals and 100 full-sibs (50 males and 50 females) of *M. nipponense* were collected from the Dapu Breeding Base in Wuxi, China (120°13′44″ E, 31°28′22″ N) (Table 3). Prior to experimentation, all prawns were acclimatized under controlled laboratory conditions for 3 days, during which water temperature was maintained at 26.0 ± 1.2 °C and dissolved oxygen was kept above 6.0 mg/L.

Muscle tissues were dissected from ten randomly selected individuals to verify the *Mn-ACTL* ORF sequence. Eighteen healthy *M. nipponense* individuals were collected for qPCR analysis. Various mature tissues, including eyestalk, brain, heart, hepatopancreas, gill, muscle, ovary, and testis, were dissected. Tissues from three individuals were pooled as one biological replicate, with six replicates prepared per tissue for qPCR. For RNAi analysis, 240 prawns (120 males and 120 females) were obtained. Muscle tissues were collected for qPCR following injection of double-stranded GFP (*dsGFP*) or double-stranded *ACTL* (*dsACTL*). Body weight and length of the full-sibs were measured, and their muscle tissue was collected for SNP identification. All samples were immediately snap-frozen in liquid nitrogen and stored at −80 °C to prevent RNA degradation.

### 4.2. Annotation and Comparison of Mn-ACTL

The full-length cDNA sequence of *Mn-ACTL* was acquired from the *M. nipponense* genome database (accession number: GCA_015104395.2) and muscle transcriptome data (accession number: SRX25177010-SRX25177021).

For sequence confirmation, total RNA was isolated from each muscle sample using RNAiso Plus reagent (TaKaRa, Dalian, China). RNA concentration was measured with a spectrophotometer (Eppendorf, Hamburg, Germany), and integrity was evaluated by agarose gel electrophoresis. Approximately 1 µg of total RNA per sample was reverse-transcribed into first-strand cDNA using the iScript™ cDNA Synthesis Kit (Bio-Rad, Hercules, CA, USA). To verify sequence accuracy, the obtained sequence was experimentally validated with three specific primer pairs (Table 4), using the synthesized cDNA as templates. The PCR products were sequenced by Shanghai Shenggong Bioengineering Technology Service Co., Ltd. (Shanghai, China) on an ABI 3730 automated DNA sequencer (Invitrogen Biotechnology Co., Ltd., Carlsbad, CA, USA).

The ORF of *Mn-ACTL* was predicted with the online tool ORF-FINDER (https://www.ncbi.nlm.nih.gov/orffinder, 15 December 2023) [35]. The corresponding cDNA sequence was translated into its amino acid sequence and visualized using DNAman software (version 6.0) [36]. Multiple sequence alignment of *ACTL* protein sequences from various species was conducted with ClustalW (version 2.0) [37]. A phylogenetic tree was subsequently constructed in MEGA (version 11) [38] based on the aligned sequences, employing the maximum likelihood method. Branch support was evaluated with 1000 bootstrap replicates, and the support values are indicated at the corresponding nodes.

### 4.3. qPCR Analysis

In the present study, the mRNA expression levels of *Mn-ACTL* in various mature tissues were quantified using qPCR. Total RNA was isolated from each tissue with RNAiso Plus Reagent (TaKaRa) according to the manufacturer’s instructions. RNA concentration was determined using a spectrophotometer (Eppendorf), and integrity was assessed by agarose gel electrophoresis. Approximately 1 µg of total RNA from each sample was reverse-transcribed into first-strand cDNA using the iScript™ cDNA Synthesis Kit (Bio-Rad).

Real-time qPCR was performed on a Bio-Rad iCycler iQ5 Real-Time PCR System using SYBR Green chemistry. The general qPCR procedures were consistent with those described in previous studies [39]. Each 25 µL reaction mixture contained 12.5 µL of 2× Ultra SYBR Mix (CWBIO, Taizhou, China), 0.5 µL of each forward and reverse primer (Table 4, 10 µM each), 1 µL of cDNA template, and 10.5 µL of PCR-grade water. The thermal cycling protocol consisted of an initial denaturation at 95 °C for 10 min, followed by 40 cycles of 95 °C for 15 s and 60 °C for 1 min. All reactions were performed in triplicate for each tissue.

The elongation factor gene (*EIF*) was employed as an internal reference [40]. The amplification efficiencies of *Mn-ACTL* and *EIF* were verified to be approximately equal, enabling the use of the 2^–ΔΔCt^ method for calculating relative gene expression [41].

### 4.4. RNAi Analysis

The potential regulatory role of *Mn-ACTL* on the growth performance of *M. nipponense* was investigated using RNAi. The 240 prawns were randomly assigned to two experimental groups: a control group injected with *dsGFP* and an experimental group injected with *dsACTL*. Each group consisted of 60 male or 60 female prawns. The *dsGFP* served as a negative control to account for non-specific effects [42]. The initial average body weight was 0.781 ± 0.11 g for males and 0.424 ± 0.05 g for females in the *dsGFP*-injected control group, and 0.781 ± 0.10 g for males and 0.417 ± 0.05 g for females in the *dsACTL*-injected group.

Gene-specific RNAi primers flanked by T7 promoter sequences were designed using Snap Dragon tools (https://www.flyrnai.org/cgi-bin/RNAi_find_primers.pl, 13 April 2024) (Table 4). Double-stranded RNA (dsRNA) targeting *Mn-ACTL* and *GFP* (control) was synthesized in vitro using the TranscriptAid™ T7 High Yield Transcription Kit (Fermentas, Inc., Waltham, MA, USA) according to the manufacturer’s instructions. Following established methods [43], prawns in the experimental and control groups were microinjected with *Mn-ACTL* dsRNA and *GFP* dsRNA (4 µg/µL each in an isotonic solution), respectively, at a dosage of 4 µg per gram of body weight. Consequently, the injection volume (in µL) administered to each prawn was numerically equivalent to its body weight (in grams). To evaluate the RNAi interference efficiency, *Mn-ACTL* mRNA expression levels in muscle tissue were analyzed by qPCR at 1, 6, 12, and 18 days post-injection (N ≥ 5 per time point). Concurrently, the body weight of each prawn was measured on the sampling days.

### 4.5. Histological Observation

Abdominal muscle samples were collected at 18 days, and histological analysis was performed to compare tissue histology between the two groups. Muscle tissues from each group were embedded using an OCT embedding kit (Rebiosci, Shanghai, China) in accordance with the manufacturer’s instructions. Longitudinal sections were prepared with an OTF5000 cryostat (Bright, San Francisco, CA, USA). For histological verification, muscle tissue samples were cryosectioned at a thickness of 10 μm. The resulting sections were examined and observed under an optical microscope (LEICA MC170 HD, Wetzlar, Germany).

### 4.6. Identification of SNPs Within ACTL

The cDNA template was synthesized via reverse transcription from total RNA isolated from the muscle tissue of each individual, as described above. The target regions were amplified by PCR with three pairs of primers using the synthesized cDNA as templates (Table 3), and the quality and specificity of the amplification products were verified by 1.2% agarose gel electrophoresis. The PCR products were then purified and subjected to bidirectional sequencing on an ABI 3730xl DNA Analyzer (Applied Biosystems, Waltham, MA, USA) by Shanghai Shenggong Bioengineering Co., Ltd. (Shanghai, China) The resulting sequences were assembled and aligned using MEGA 11.0 software [38] to identify SNP loci within the *ACTL* gene, according to a previous study [33,34]. The associations between the identified SNP loci and growth traits (body weight and total length) were analyzed using SPSS Statistics 23.0, with these traits treated as dependent variables.

### 4.7. Statistical Analysis

Statistical analyses were conducted using SPSS Statistics 23.0. Significant differences among groups were evaluated by one-way ANOVA, supplemented with LSD and Duncan’s post hoc tests for multiple comparisons. The results from quantitative data were expressed as mean ± standard deviation (SD), and a *p*-value of less than 0.05 was regarded as statistically significant.

## 5. Conclusions

Actin is a known regulator of crustacean growth. Our results support this role by showing that *ACTL* positively influences growth performance in both sexes of *M. nipponense*. Moreover, growth-associated SNPs identified within the *ACTL* gene promote the use of marker-assisted selection for genetic breeding in this species.

## Figures and Tables

**Table 1:** Identification of SNPs within the *Mn-ATCL*.

SNP	Genotype 1	Genotype 2	Genotype 3	*Ho*	*He*	*PIC*	FDR	Type
S28_17145758	C: 65	G: 2	S: 24	0.263	0.260	0.226	0.044	Synonymous
S28_17147514	C: 60	T: 5	Y: 32	0.330	0.339	0.282	0.671	Synonymous
S28_17147694	C: 55	T: 4	Y: 39	0.398	0.365	0.298	0.283	Synonymous
S28_17147736	C: 52	T: 5	Y: 41	0.418	0.385	0.311	0.329	Synonymous
S28_17147856	C: 63	T: 1	Y: 33	0.340	0.296	0.252	0.170	Synonymous
S28_17147898	C: 71	T: 1	Y: 26	0.265	0.245	0.215	0.162	Synonymous
S28_17147904	A: 1	G: 71	R: 26	0.265	0.245	0.215	0.162	Synonymous
S28_17147928	A: 78	C: 2	M: 17	0.175	0.193	0.174	0.421	Synonymous
S28_17147940	A: 84	T: 1	W: 12	0.124	0.134	0.125	0.516	Synonymous
S28_17147967	C: 3	T: 85	Y: 7	0.074	0.127	0.119	0.217	Synonymous
S28_17148114	A: 69	G: 3	R: 25	0.258	0.269	0.232	0.588	Synonymous
S28_17148150	C: 3	T: 73	Y: 19	0.200	0.229	0.202	0.989	Synonymous
S28_17148225	C: 70	T: 2	Y: 25	0.258	0.254	0.222	0.516	Synonymous
S28_17148432	C: 6	T: 63	Y: 26	0.274	0.320	0.269	0.097	Synonymous
S28_17148483	C: 6	T: 72	Y: 17	0.179	0.259	0.225	0.592	Synonymous
S28_17148489	C: 72	T: 8	Y: 15	0.158	0.273	0.236	0.621	Synonymous
S28_17148573	C: 6	T: 62	Y: 25	0.269	0.319	0.268	0.593	Synonymous
S28_17149891	A: 68	T: 5	W: 13	0.151	0.231	0.204	0.049	Synonymous

**Table 2:** Identification of growth-associated SNPs within the *Mn-ATCL*.

SNP ID	Gender	Genotype (Number)	Weight (g)	Full Length (mm)
S28_17145758	All	CC: 65	1.336 ± 0.396 a	43.690 ± 6.157 a
		CG: 24	1.668 ± 0.509 a	52.403 ± 5.747 b
		GG: 2	2.585 ± 0.403 b	60.375 ± 0.262 c
	Female	CC: 34	0.836 ± 0.396 a	42.690 ± 6.157 a
		CG: 11	1.268 ± 0.509 b	48.403 ± 5.747 b
		GG: 0	/	/
	Male	CC: 31	1.781 ± 0.914 a	53.710 ± 8.768 a
		CG: 13	2.071 ± 0.916 a	56.285 ± 7.941 a
		GG: 2	2.585 ± 0.403 b	60.375 ± 0.262 b
S28_17149891	All	AA: 68	1.338 ± 0.384 a	48.720 ± 5.790 a
		AT: 13	1.245 ± 0.518 a	47.750 ± 8.360 a
		TT: 5	2.020 ± 0.537 b	56.375 ± 6.951 b
	Female	AA: 38	0.838 ± 0.384 a	42.720 ± 5.790 a
		AT: 4	0.930 ± 0.518 a	44.750 ± 8.360 a
		TT: 2	1.520 ± 0.537 b	50.375 ± 6.951 a
	Male	AA: 30	1.892 ± 0.913 a	54.742 ± 8.456 a
		AT: 9	1.559 ± 0.868 a	51.744 ± 8.370 a
		TT: 3	2.493 ± 0.150 b	61.047 ± 2.287 b

Lowercase letters indicated the significant difference between different genotypes (*p* < 0.05).

**Table 3:** Specimens used in this study.

Sampling Data	Animals	Tissue	Purpose
4–7 July 2023	10 specimens	Muscle	ORF verification
4–7 July 2023	18 specimens	Eyestalk, Brain, Heart, Hepatopancreas, Gill, Muscle, Ovary, Testis	qPCR analysis
15 June–6 July 2024	240 specimens (120 males and 120 females)	Muscle	RNAi analysis
12 September 2024	100 specimens (50 males and 50 females) from a full-sib family	Muscle	SNP identification

**Table 4:** Primers used in the present study.

Primer	Sequence	Purpose
F1	CATTTGGACTCCGACAGGGA	Primers for PCR verification and SNP identification
R1	TAAGTGGCGGGCATGTTGAA	
F2	TCGAGTCCTTCAACATGCCC	
R2	GTCGCACCTCATGACAGAGT	
F3	GAGCTTCCTGATGGTCAGGTT	
R3	TTGCTTAGAAGCACTTGCGG	
RT-F1	TCTGTCATGAGGTGCGACAT	Primer for qPCR
RT-F2	CTTCTGCATCCTGTCAGCAA	
EIF-F1	CATGGATGTACCTGTGGTGAAAC	Primer for reference gene
EIF-R1	CTGTCAGCAGAAGGTCCTCATTA	
RNAi-F1	TAATACGACTCACTATAGGGTCTGTCATGAGGTGCGACAT	Primer for RNAi
RNAi-R1	TAATACGACTCACTATAGGGCTTCTGCATCCTGTCAGCAA	

## Data Availability

The raw data supporting the conclusions of this article will be made available by the authors on request.
